# Oleil Hydroxytyrosol (HTOL) Exerts Anti-Myeloma Activity by Antagonizing Key Survival Pathways in Malignant Plasma Cells

**DOI:** 10.3390/ijms222111639

**Published:** 2021-10-28

**Authors:** Katia Todoerti, Maria Eugenia Gallo Cantafio, Manuela Oliverio, Giada Juli, Carmine Rocca, Rita Citraro, Pierfrancesco Tassone, Antonio Procopio, Giovambattista De Sarro, Antonino Neri, Giuseppe Viglietto, Nicola Amodio

**Affiliations:** 1 Department of Oncology and Hemato-Oncology, University of Milano, 20122 Milano, Italy; katia.todoerti@unimi.it (K.T.); antonino.neri@unimi.it (A.N.); 2 Hematology Unit, Fondazione Cà Granda IRCCS, Ospedale Maggiore Policlinico, 20122 Milano, Italy; 3 Department of Experimental and Clinical Medicine, Magna Graecia University of Catanzaro, 88100 Catanzaro, Italy; mariaeugenia.gallocantafio@unicz.it (M.E.G.C.); giadajuli@libero.it (G.J.); tassone@unicz.it (P.T.); viglietto@unicz.it (G.V.); 4 Department of Health Sciences, Magna Graecia University of Catanzaro, 88100 Catanzaro, Italy; m.oliverio@unicz.it (M.O.); citraro@unicz.it (R.C.); desarro@unicz.it (G.D.S.); 5 Laboratory of Cellular and Molecular Cardiovascular Physiology, Department of Biology, Ecology and Earth Sciences (Di.B.E.S.T.), University of Calabria, 87036 Rende, Italy; carmine.rocca@unical.it

**Keywords:** experimental therapeutics, multiple myeloma, natural anti-tumor agents, oleil hydroxytyrosol

## Abstract

Polyphenols from olive oil are endowed with several biological activities. Chemical modifications have been recently applied to these compounds to improve their therapeutic activity in different pathological settings, including cancer. Herein, we describe the in vitro effects on multiple myeloma (MM) cells of oleil hydroxytyrosol (HTOL), a synthetic fatty ester of natural hydroxytyrosol with oleic acid. HTOL reduced the viability of various human MM cell lines (HMCLs), even when co-cultured with bone marrow stromal cells, triggering ER stress, UPR and apoptosis, while it was not cytotoxic against healthy peripheral blood mononuclear cells or B lymphocytes. Whole-transcriptome profiling of HTOL-treated MM cells, coupled with protein expression analyses, indicate that HTOL antagonizes key survival pathways for malignant plasma cells, including the undruggable IRF4–c-MYC oncogenic axis. Accordingly, c-MYC gain- and loss-of-function strategies demonstrate that HTOL anti-tumor activity was, at least in part, due to c-MYC targeting. Taken together, these findings underscore the anti-MM potential of HTOL, providing the molecular framework for further investigation of HTOL-based treatments as novel anti-cancer agents.

## 1. Introduction

Multiple myeloma (MM) is a B cell malignancy of clonal plasma cells (PCs) accumulating in the bone marrow (BM), characterized by a complex genomic and epigenomic landscape [1,2,3]. Despite remarkable therapeutic advancements that have led to increased extent and frequency of response with unprecedented outcome improvement, MM patients eventually evolve towards a fatal drug-resistant phase [2,4].

Scientific interest in natural drugs and their derivatives has recently grown due to the limitations and toxicity of conventional therapies in comparison to the safety of natural-product-derived compounds. In this regard, the biological activities of plant extracts have been extensively linked to their content in various molecules, such as polyphenols, flavonoids and terpenoids, known to elicit therapeutic effects against cancer, cardiovascular and neurodegenerative diseases. Moreover, the combination of plant extracts with anti-cancer drugs has often shown synergistic therapeutic efficacy by sensitizing malignant cells to chemotherapy, and/or overcoming drug resistance in various cancer types [5,6], including MM [7,8,9].

A major source of anti-tumor polyphenols is extra virgin olive oil (EVOO), the polyphenolic fraction of which includes simple phenols (tyrosol and hydroxytyrosol), secoiridoids (oleuropein, oleocanthal and oleacein) and lignans [9,10,11,12,13].

Hydroxytyrosol (2-(3,4-dihydroxyphenyl)ethanol; HT) belongs to the family of ortho-diphenolic compounds, derived from the hydrolysis of the secoiridoid oleuropein, and displays an amphiphilic character. HT acts as a natural antioxidant due to its catechol moiety, and assumes a protective role especially in bulk oils; moreover, HT has been largely investigated owing to its beneficial effects on human health, particularly in relation to cardiovascular diseases, microbial infections and cancer [14,15,16].

To envisage an extensive use of HT in food, cosmetics and pharmaceuticals, more lipophilic HT ethers or esters have been synthesized, which display remarkable antioxidant activity in oil/water emulsions, especially when the elongation of the chain is comprises between C4 and C8 atoms [17,18]. Longer chains (C10−C16) have enabled new pharmaceutical applications of HT, including its integration in liposomes for topical administration [19,20]. Overall, these studies demonstrate that the biological activity of lipophilic derivatives is higher than free HT.

Oleil hydroxytyrosol (HTOL), a synthetic fatty ester of natural HT with oleic acid, is endowed with a significant lipophilicity by preserving the catechol moiety of HT, which is mainly responsible for its biological activities [21,22]. Such a chemical modification can be realized following very mild procedures for the synthesis of HT esters [16,20]. The obtained HTOL appears as a good candidate in formulations for the treatment of cutaneous infectious diseases [20]; moreover, it displayed antimicrobial activities against *S. aureus* and *S. epidermidis* [23]. Conversely, its anti-tumor potential has never been investigated. Here, we analyzed the anti-tumor effects and the molecular perturbations produced by HTOL treatment in MM cells. Taken together, our findings highlight, for the first time, the significant anti-MM effects of HTOL in vitro through the targeting of molecular pathways key for malignant plasma cell survival.

## 2. Results

### 2.1. HTOL Exerts Anti-Tumor Activity in HMCLs

**Figure 1:**
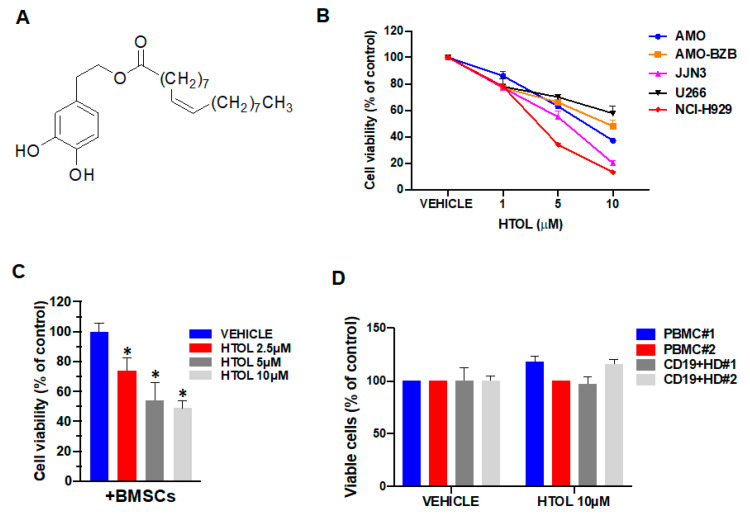
HTOL effects on HMCLs and PBMCs viability. (**A**) Chemical structure of HTOL. (**B**) Cell viability of HMCLs as determined by Cell Titer Glo (CTG) assay 48 h after treatment with increasing doses of HTOL or vehicle (DMSO). (**C**) Luciferase-engineered, GFP-positive NCI-H929 cells were plated adherent to MM BMSCs and treated for 48 h with HTOL;cell viability was determined by luciferase assay and expressed as a percentage of the luciferase activity of vehicle-treated cells; * *p* < 0.05 as compared to vehicle-treated cells. (**D**) CTG assay performed on peripheral blood mononuclear cells (PBMCs) or on purified CD19^+^ cells from two different healthy donors (HD), treated ex vivo with HTOL for 48 h.

HTOL, the chemical structure of which is reported in Figure 1A, was obtained by a standardized procedure as reported in Materials and Methods.

To evaluate the potential anti-tumor activity of HTOL in vitro, we used five different HMCLs, reflecting, at least partially, the genetic heterogeneity of the disease and including bortezomib-resistant cells (AMO-BZB) that mimic a refractory disease. Treatment for 48 h with increasing doses of HTOL reduced HMCLs viability, with an IC50 ranging from 5.0 to 10.0 μM (Figure 1B; Supplementary Figure S1). We next tested the in vitro anti-tumor activity of HTOL in MM cells co-cultured with MM patient-derived bone marrow stromal cells (BMSCs), known to sustain growth and to promote the chemoresistance of MM cells. Luciferase-engineered GFP-expressing NCI-H929 cells were cultured adherent to malignant BMSCs, and then treated for 48 h with HTOL. The results indicate that HTOL dampened MM cell viability even in co-culture conditions, thus overcoming the protective effects of BMSCs (Figure 1C).

Finally, we evaluated whether HTOL might be cytotoxic against non-tumor cells. To this aim, we tested its effects on both healthy PBMCs or B lymphocytes. It is noteworthy that no cytotoxicity was detected in these cells, isolated from two different healthy donors and treated ex vivo with HTOL, underscoring a favorable therapeutic window for this compound (Figure 1D).

### 2.2. Transcriptional Signatures and Molecular Pathways Modulated by HTOL Treatment in MM

To uncover the biological scenario underlying its cytotoxicity against HMCLs, the specific transcriptional signature and molecular pathways affected by HTOL treatment were evaluated by GSEA analysis of the global protein-coding gene expression profiles of HTOL-treated versus vehicle-treated JJN3 cells. Gene sets involved in cell cycle regulation, apoptosis and DNA and RNA metabolism were found to be positively modulated amongst others, whereas genes of the Interferon Gamma response were found to be significantly down-regulated upon HTOL exposure (Supplementary Table S1).

### 2.3. HTOL Triggers ER Stress, UPR and Apoptosis

**Figure 2:**
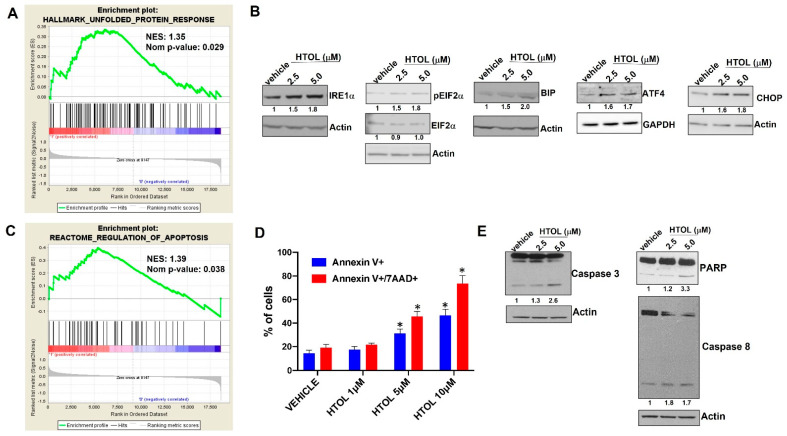
HTOL induces ER stress, UPR and apoptosis of JJN3 cells. (**A**) GSEA performed 24 h after treatment of JJN3 cells with 5.0 μM HTOL shows enrichment of UPR genes; normalized enrichment score (NES) and nominal *p*-value parameters are reported. (**B**) Western blot (WB) analysis of IRE1α, phospho-EIF2α, EIF2α, BIP, ATF4 and CHOP in JJN3 whole cell lysates after HTOL treatment for 24 h; actin or GAPDH were used as loading control. (**C**) GSEA performed 24 h after treatment of JJN3 cells with 5.0 μM HTOL shows enrichment of apoptotic genes; normalized enrichment score (NES) and nominal *p*-value parameters are reported. (**D**) Annexin V/7-AAD staining of JJN3 cells after treatment with HTOL for 24 h. (**E**) WB of caspase-3, caspase-8 and PARP1 in JJN3 cells after 24 h of HTOL treatment; actin was used as loading control. * *p* < 0.05 as compared to vehicle-treated cells.

The elevated production and secretion of paraproteins by MM cells induce ER stress which, in turn, activates the unfolded protein response (UPR). Interestingly, we found the “UPR gene set” (Hallmark collection) amongst the most significantly and positively modulated gene sets at a lower stringency level (nom. *p*-value < 0.05) upon HTOL treatment (Figure 2A). Accordingly, WB analysis demonstrated that HTOL increased phosphorylation of eIF2α, and induced the expression of ER stress sensor proteins such as inositol-requiring enzyme 1α (IRE1α), the chaperone BiP/GRP78 (Figure 2C), the Activating Transcription Factor 4 (ATF4) and the UPR-induced proapoptotic CCAAT/enhancer-binding protein (C/EBP) homologous protein (CHOP), supporting the idea that ER-stress-induced UPR is a pro-death mechanism.

As a further demonstration of its pro-apoptotic activity, we identified by FACS analysis a dose-dependent increase in Annexin-V positive and Annexin-V/7-AAD double positive cells triggered by HTOL, suggestive of early and late apoptosis occurrence, respectively (Figure 2D). Similar results were obtained in NCI-H929 cells (Supplementary Figure S2). In line with these findings, “Reactome regulation of apoptosis” was among the positively modulated gene sets after HTOL treatment (nom. *p*-value < 0.05; Figure 2D). Accordingly, within the core enrichment genes we observed a significant positive modulation of caspase genes (i.e., CASP3, CASP9) (Supplementary Figure S3). Finally, WB analysis of HTOL-treated JJN3 cells confirmed the activation of caspase-3, caspase-8 and PARP, thus corroborating the pro-apoptotic effects of HTOL (Figure 2E).

Overall, these results suggest that ER-stress-induced apoptosis may contribute to HTOL cytotoxicity.

### 2.4. HTOL Negatively Regulates the Undruggable IRF4 Pathway in HMCLs

**Figure 3:**
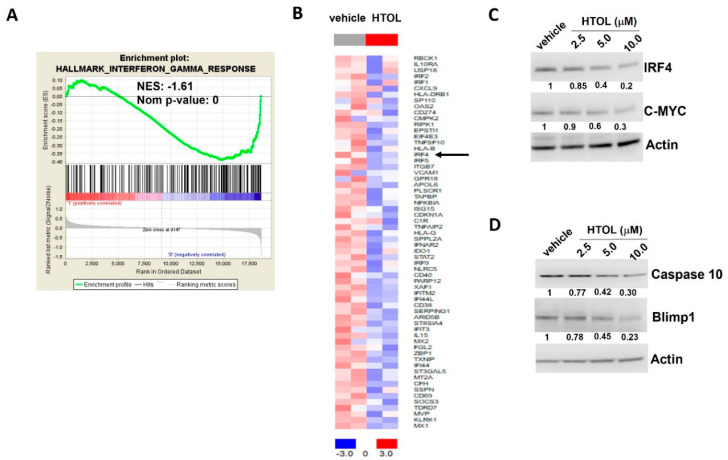
HTOL inhibits the IRF4 pathway. (**A**) GSEA performed 24 h after treatment of JJN3 cells with 5.0 μM HTOL shows a decrease in IFN-γ genes in JJN3 cells. (**B**) Heatmap analysis reporting the core enrichment genes of the IFN-γ pathway, also including IRF4, found them to be significantly down-regulated after 5.0 μM HTOL treatment of JJN3 cells. WB of (**C**) IRF4, c-MYC, (**D**) caspase-10 and Blimp1 in JJN3 cells exposed for 24 h to HTOL treatment; actin was used as loading control.

Intriguingly, our GSEA analysis indicates the “Hallmark of Interferon Gamma response” as the only gene set down-regulated by HTOL in JJN3 cells (Figure 3A). Within the core gene set, we observed a down-regulation of the lymphocyte-specific transcription factor Interferon regulatory factor 4 (IRF4) mRNA (Figure 3B); it encodes a key transcriptional regulator of such pathway and is considered an “Achilles’ heel” in MM, since its targeting is lethal for MM cells, irrespective of their genetics. Accordingly, WB analysis showed that HTOL treatment resulted in dose-dependent down-regulation of IRF4 protein, along with reduced protein levels of relevant IRF4 target genes, such as c-MYC, caspase-10 and Blimp1, in JJN3 (Figure 3C,D), as well as in NCI-H929 cells (Supplementary Figure S4). Overall, these results indicate that HTOL treatment impacts on the oncogenic IRF4 signaling in MM cells.

### 2.5. c-MYC Affects HTOL Sensitivity of HMCLs

The IRF4 target c-MYC is overexpressed during MM progression and plays a critical role in drug resistance to conventional and novel anti-MM therapies [24,25]. By orchestrating a pro-cancer program across multiple cellular pathways, c-MYC acts as a fundamental positive regulator of MM cell growth [26]. Given the relevance of c-MYC in MM pathobiology and based on the above-reported results on the HTOL-mediated down-modulation of IRF4 targets, by using both gain- and loss-of-function approaches, we sought to investigate whether c-MYC levels affect the HTOL sensitivity of HMCL.

**Figure 4:**
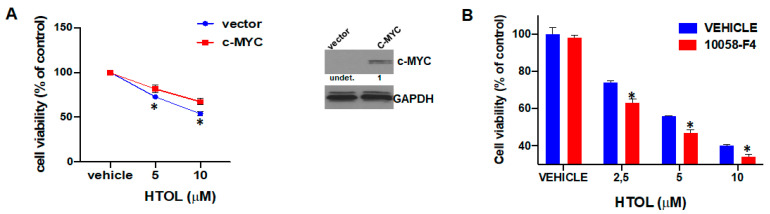
c-MYC affects MM cells’ responsiveness to HTOL. (**A**) Cell viability of U266 transduced with pCDH empty vector or pCDH-c-MYC as determined by CTG assay, 24 h after treatment with increasing doses of HTOL or vehicle (DMSO); * *p* < 0.05 as compared to empty-vector transfected cells. Right panel refers to WB analysis of c-MYC performed using lysates from U266 cells transduced with pCDH empty vector or pCDH–cMYC. (**B**) Cell viability as determined by CTG assay in NCI-H929 cells treated for 24 h with 10 μM 10058-F4 in combination with HTOL or DMSO; * *p* < 0.05 as compared to vehicle-treated cells.

It is noteworthy that enforced expression of c-MYC in the c-MYC-null U266 cell line significantly antagonized HTOL inhibitory effects on cell viability (Figure 4A); conversely, c-MYC inhibition in NCI-H929 cells by 10058-F4 compound slightly increased HTOL sensitivity (Figure 4B). Collectively, these data demonstrate that c-MYC targeting might contribute, at least in part, to the inhibitory effects exerted by HTOL on HMCL viability.

## 3. Discussion

Various natural compounds obtained from different sources have been shown to prevent the onset of primary cancers, or to antagonize the evolution of pre-malignant and malignant conditions towards more aggressive stages. It is noteworthy that recent preclinical studies have also demonstrated the significant advantages of many of these agents for the management of MM [7], with a therapeutic window even superior to conventional chemotherapeutics. Mechanistically, the inhibition of oncogenic signaling pathways and the modulation of the epigenome have been found to be directly associated with the anti-tumor activity of such compounds [7,27]. Polyphenols found in olive oil have been found to be protective in various disease contexts [22]. In this regard, the proactive ingredient oleuropein and its derivative HT have been widely studied over the last decade, demonstrating many beneficial effects in cancer preclinical models [28].

We recently synthetized HTOL, a synthetic fatty ester of natural HT with oleic acid [21,22], through very mild synthetic procedures [16,20]. In the present work, we investigated for the first time the potential anti-tumor activity and the molecular pathways affected by HTOL in MM cells. Interestingly, our data demonstrate that HTOL reduces, at low micromolar doses, the viability of HMCLs, even in the presence of BMSCs, without being cytotoxic to healthy PBMCs or B lymphocytes, providing the basis for further investigation of HTOL activity using in vivo models of the disease.

Additionally, by whole-transcriptome analysis, we studied the molecular perturbations and the functional consequences induced by HTOL treatment in vitro. Gene ontology analysis revealed that genes associated with ER stress, UPR and apoptosis are induced by HTOL treatment, supporting the notion that HTOL-mediated cytotoxicity could likely be ascribed to the activation of the aforementioned pathways.

In parallel, our analysis revealed a down-regulation of Interferon-γ signaling produced by HTOL. Interferon regulatory factors (IRFs) are a family of transcription factors that regulate many aspects of innate and adaptive immune responses—including anti-viral responses, pro-inflammatory responses against pathogens and regulation of immune cell differentiation [29]. Comprising nine family members, the IRFs share significant homology within their N-terminal DNA-binding domain (DBD), which forms a helix-loop-helix motif that recognizes specific DNA sequences similar to the Interferon-stimulated response element. Interferon regulatory factor 4 (IRF4) acts as a lymphocyte-specific transcription factor [30]; it is oncogenic and overexpressed when translocated to actively transcribed genomic regions in some MM patients, but it also has a survival effect in MM in the absence of translocations or amplification [30]. We, and others, have demonstrated that IRF4 genetic knockdown, or its targeting via the tumor suppressor miR-125b, is lethal for MM cells, irrespective of their genetics, making IRF4 a sort of “Achilles’ heel” for MM [31,32]. A downstream IRF4 effector is the B-lymphocyte-induced maturation protein-1 (BLIMP-1) [33], the knockdown of which causes apoptosis in MM cells. Moreover, it was recently demonstrated that caspase-10 is transactivated by IRF4; importantly, the recent evidence by Lamy et al. that all HMCLs require casp-10 for survival has led to the assumption that loss of the casp-10/cFlip heterodimer mediates MM cell death induced by IRF4 knockdown [34]. All these data make IRF4 an attractive therapeutic target in MM [31].

A further relevant IRF4 target gene is c-MYC, which has a prominent role in the pathogenesis of MM [31,35]. c-MYC is overexpressed during MM progression, with the highest levels detected in plasma cell leukemias. Furthermore, c-MYC plays a critical role in drug resistance to conventional anti-MM therapy, such as melphalan, as well as to immunomodulatory drugs [24,25]. Intriguingly, our findings indicate that HTOL is able to antagonize the IRF4 pathway, leading to a significant down-regulation of IRF4 and its validated targets, which likely contributes to its anti-MM activity in our experimental models. Moreover, we applied c-MYC gain- and loss-of-function strategies and demonstrated that the manipulation of c-MYC affected, at least partially, HTOL sensitivity, further strengthening the capability of HTOL to act by antagonizing such oncogenic pathway. Since the IRF4–c-MYC axis is overexpressed in MM cells, it is likely that the lack of toxicity of HTOL on healthy cells can be ascribed to the absence or low expression of its targets.

It is of note that the IRF4–c-MYC axis remains undruggable since no targeted therapies are currently available and clinically exploitable to selectively inhibit these targets. It is tempting to speculate that HTOL-based therapeutics, through the inhibition of these potent oncogenic drivers, might provide new options applicable not only to MM but also to other c-MYC-addicted cancer types. Future studies are planned to assess HTOL anti-tumor effects in the context of in vivo MM preclinical models, along with the appropriate pharmacokinetics studies that are required for translating these findings in humans.

## 4. Materials and Methods

### 4.1. Chemicals

HTOL was obtained by esterification of hydroxytyrosol with oleyl chloride according to a previously described procedure [20]. Briefly, equimolar quantities of HT and oleyl chloride were dissolved in dry THF at room temperature, in the presence of 1% mol of Er(OTf)_3,_ and underwent reaction for 12 h. The product was purified after work-up by flash chromatography. The c-MYC inhibitor 10058-F4 (F3680) was from Merck.

### 4.2. Cell Cultures

NCI-H929, U266 and JJN3 human multiple myeloma cell lines (HMCLs) were purchased from DSMZ, which certified authentication performed by short tandem repeat DNA typing. AMO-1 and bortezomib-resistant AMO-BZB cells were kindly provided by Dr. C. Driessen (University of Tubingen, Germany). All these cell lines were immediately frozen and used from the original stock within 6 months. HMCLs were cultured in RPMI-1640 media containing 10% FBS (GIBCO; Life Technologies, Carlsbad, CA, USA), 2 μmol/L glutamine, 100 U/mL penicillin and 100 μg/mL streptomycin (GIBCO; Life Technologies, Carlsbad, CA, USA) and tested for mycoplasma contamination. To generate U266 cells stably expressing c-MYC, parental U266 cells were transduced with Precision LentiORF human MYC (GE Dharmacon, Lafayette, CO, USA) or empty vector, as previously reported [36]. Peripheral blood mononuclear cells (PBMCs) from healthy donors’ buffy coats were isolated by Ficoll-hypaque (Lonza Group, Basel, Switzerland), in accordance with the Declaration of Helsinki following informed consent and Institutional Review Board (University of Catanzaro, Catanzaro, Italy) approval (institutional approval: n.120/2015), as previously reported [37]. CD19^+^ cells were obtained from PBMCs using an anti-human anti-CD19 microbeads kit (Milteny Biotech, Gladbach, Germany) according to the manufacturer’s instructions. NCI-H929 cells stably expressing luciferase and GFP transgenes were obtained by transducing NCI-H929 cells with pLentipuro3/TO/V5-GE/EGFP-Firefly luciferase lentivirus (Addgene), as previously reported [36]. BMSCs were obtained from a BM aspirate of a newly diagnosed MM patient following depletion of CD138^+^ cells using an anti-human CD138 microbeads kit (Milteny Biotech, Gladbach, Germany). Co-culture experiments were performed in 96-well plates at a density of 4.0 × 10^4^ NCI-H929 GFP-luciferase-positive cells/mL in a 2:1 BMSCs/MM cells ratio. Cell viability was determined in vehicle- or HTOL-treated co-cultures by measuring the luminescence of luciferase-expressing cells with the luciferase reporter assay (Promega) and reported as % of the untreated.

### 4.3. Cell Viability and Apoptosis Assays

Cell viability was analyzed by Cell Titer Glo (CTG; Promega), while Annexin V/7-Aminoactinomycin (7-AAD) flow cytometry assay (BD Biosciences, San Jose, CA, USA) was used to define apoptosis and cell viability according to the manufacturer’s instructions.

### 4.4. Western Blotting

Whole-cell protein extracts were prepared using NP40 containing Halt Protease Inhibitor cocktail (Invitrogen, Thermo Fisher Scientific, Waltham, MA, USA). Western blot (WB) was performed as reported [9]. Antibodies were all from Cell Signaling Technology (anti-PARP (#9532), -CHOP (#2895), -ATF-4 (#11815), -IREα (#3294), -Phospho-eIF2α (#3398), -eIF2α (#9722), -BIP (#3177), -caspase-8 (#9746), -caspase-3 (#9665), -IRF4(#4694), -caspase-10 (#9752), Blimp1 (#9115), -β-actin (#3700), -c-MYC (#5605) and GAPDH (#97166)). Densitometric analysis of blots was performed by LI-COR Image Studio Digits Ver 5.0 (Bad Homburg, Germany) and expressed as a relative protein unit after normalization with appropriate housekeeping; a representative blot, with the relative densitometric analysis, is shown in each figure.

### 4.5. Transcriptional Profiling by Microarray

Global gene expression profiles of duplicate JJN3 DMSO-treated control cells and cells treated for 24 h with 5.0 μM HTOL were obtained by means of GeneChip^®^ Human Gene 2.0 ST arrays (Thermo Fisher), as previously described [38]. RNA was obtained using Trizol reagent (Invitrogen). The expression levels of Ensembl genes specific for 18,642 unique protein-coding genes were obtained and compared by Gene Set Enrichment Analysis (GSEA). Default analysis conditions and 500 gene set permutations were applied on gene sets (5–500 genes) of Kegg, Reactome and Hallmark collections. The most significant gene sets were selected on the basis of nominal *p*-value (<0.05) and FDR q-value (<10%). Gene expression data were deposited in the Gene Expression Omnibus (GEO) repository under GEO accession number GSE184489.

### 4.6. Statistical Analysis

Each experiment was performed at least three times, and values were reported as mean ± SD. Data were analyzed using Student’s *t* tests for two-group comparisons or a one-way analysis of variance (ANOVA) for multiple comparisons using the Graphpad software (GraphPad Software, La Jolla, CA, USA). A *p*-value < 0.05 was considered significant.

## Data Availability

The data presented in this study are available in this article (and the supplementary material).

## Supplementary Materials

The following are available online at https://www.mdpi.com/article/10.3390/ijms222111639/s1.
