# Supplementary material for: Oleil Hydroxytyrosol (HTOL) Exerts Anti-Myeloma Activity by Antagonizing Key Survival Pathways in Malignant Plasma Cells
Source: Int J Mol Sci. 2021 Oct 28;22(21):11639. doi: 10.3390/ijms222111639 (PMC8584245; doi:10.3390/ijms222111639)
Supplement: Supplementary file 1 [file ijms-22-11639-s001.zip › ijms-1409726-supplementary.pdf]

Table S1: List of the most significant (nom. p-val<0.05 and FDR q-val<10%) gene sets up- or down-regulated in HTOL treated versus control JJN3 duplicates. Normalized Enrichment score (NES), nominal p-value, FDR q-value are reported for each KEGG, REACTOME or HALLMARK gene set. Gene sets are ordered according to nominal p-value in each collection.

NAME	SIZE	NES	NOM p-val	FDR q-val
KEGG_DNA_REPLICATION	36	1,8629572	0	0,0396558
REACTOME_G2_M_CHECKPOINTS	41	2,1805267	0	0,0010061
REACTOME_ACTIVATION_OF_ATR_IN_RESPONSE_TO_REPLICATION_STRESS	35	2,120249	0	0,0010927
REACTOME_DNA_REPLICATION	182	2,1445453	0	0,0014569
REACTOME_MITOTIC_M_M_G1_PHASES	162	2,2236993	0	0,0020122
REACTOME_CELL_CYCLE_CHECKPOINTS	110	2,0874388	0	0,0022694
REACTOME_M_G1_TRANSITION	77	2,0318806	0	0,0041646
REACTOME_ACTIVATION_OF_THE_PRE_REPLICATIVE_COMPLEX	30	2,0294888	0	0,0042194
REACTOME_CELL_CYCLE_MITOTIC	300	1,9518813	0	0,0153273
REACTOME_PROCESSING_OF_INTRONLESS_PRE_MRNAS	14	1,8719659	0	0,0225047
REACTOME_MITOTIC_G1_G1_S_PHASES	128	1,8755218	0	0,0228139
REACTOME_ENDOSOMAL_SORTING_COMPLEX_REQUIRED_FOR_TRANSPORT_ESCRT	25	1,8801295	0	0,0236449
REACTOME_SYNTHESIS_OF_DNA	89	1,8839159	0	0,0247668
REACTOME_MITOTIC_PROMETAPHASE	81	1,8577735	0	0,0256893
REACTOME_S_PHASE	105	1,8848453	0	0,0266455
REACTOME_CELL_CYCLE	378	1,8872088	0	0,0272056
REACTOME_G1_S_TRANSITION	104	1,8963621	0	0,0300775
REACTOME_FACILITATIVE_NA_INDEPENDENT_GLUCOSE_TRANSPORTERS	12	1,8120723	0	0,036673
REACTOME_DNA_STRAND_ELONGATION	30	1,8126976	0	0,0383447
REACTOME_ASSEMBLY_OF_THE_PRE_REPLICATIVE_COMPLEX	62	1,7475202	0	0,0583408
REACTOME_HIV_LIFE_CYCLE	107	1,6933157	0	0,0878187
REACTOME_CLEAVAGE_OF_GROWING_TRANSCRIPT_IN_THE_TERMINATION_REGION_	40	1,6951902	0	0,0895878
REACTOME_UNWINDING_OF_DNA	11	1,7596835	0,004273505	0,05566
REACTOME_MRNA_3_END_PROCESSING	32	1,6907833	0,004366812	0,0842613
REACTOME_G0_AND_EARLY_G1	23	1,7261058	0,004484305	0,0687357
REACTOME_FANCONI_ANEMIA_PATHWAY	19	1,7840939	0,004504505	0,0491063
REACTOME_FORMATION_OF_TUBULIN_FOLDING_INTERMEDIATES_BY_CCT_TRIC	21	1,8906984	0,004524887	0,0286232
REACTOME_EXTENSION_OF_TELOMERES	27	1,7738888	0,004545454	0,0528074

**REACTOME_E2F_ENABLED_INHIBITION_OF_PRE_REPLICATION_COMPLEX_FORMATION**	10	1,712782	0,008230452	0,077351
**REACTOME_ASSOCIATION_OF_LICENSING_FACTORS_WITH_THE_PRE_REPLICATIVE_COMPLEX**	13	1,7695241	0,008438818	0,0526726
**REACTOME_PROCESSING_OF_CAPPED_INTRONLESS_PRE_MRNA**	22	1,7539661	0,00877193	0,0571675
**REACTOME_FORMATION_OF_THE_TERNARY_COMPLEX_AND_SUBSEQUENTLY_THE_43S_COMPLEX**	45	1,6914027	0,009049774	0,0866961
**HALLMARK_E2F_TARGETS**	197	2,309019	0	0
**HALLMARK_G2M_CHECKPOINT**	194	1,8165199	0	6,18E-04
**HALLMARK_INTERFERON_GAMMA_RESPONSE**	196	-1,6052411	0	0,0754253

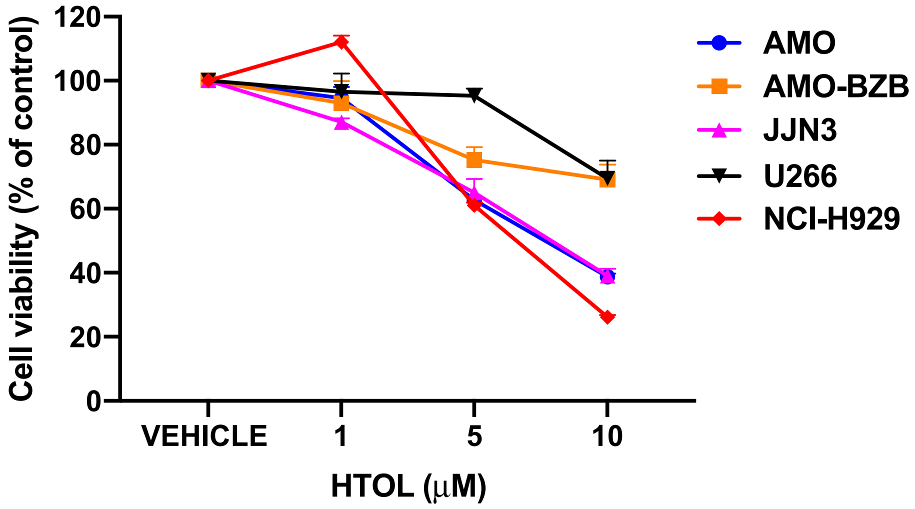

Figure S1: Cell viability of HMCLs as determined by Cell Titer Glo (CTG) assay 24 h after treatment with increasing doses of HTOL or vehicle (DMSO).

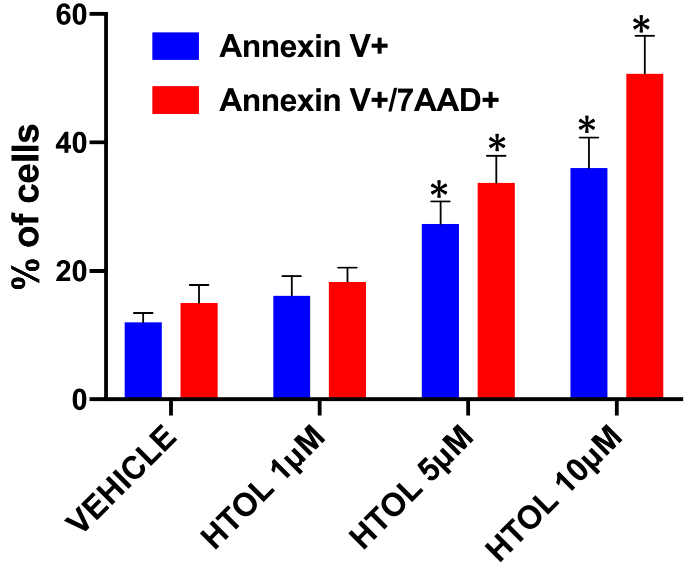

Figure S2: Annexin V/7-AAD staining of NCI-H929 cells after treatment with HTOL for 24 h. * p< 0.05 as compared to vehicle-treated cells.

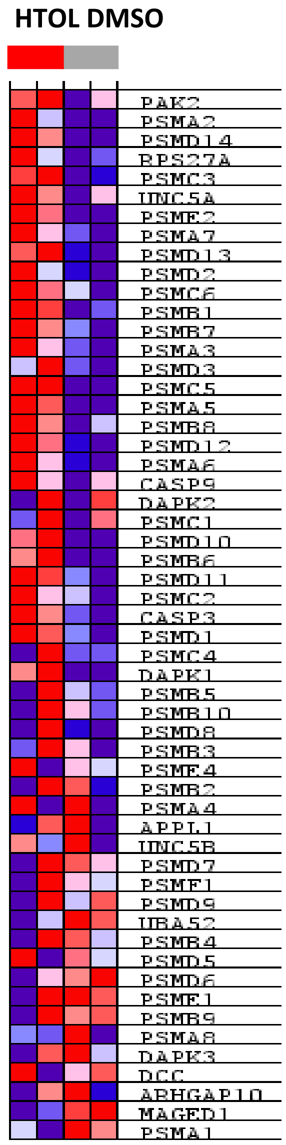

Figure S3: Heatmap of the core enrichment genes for "Reactome Regulation of Apoptosis" gene set.

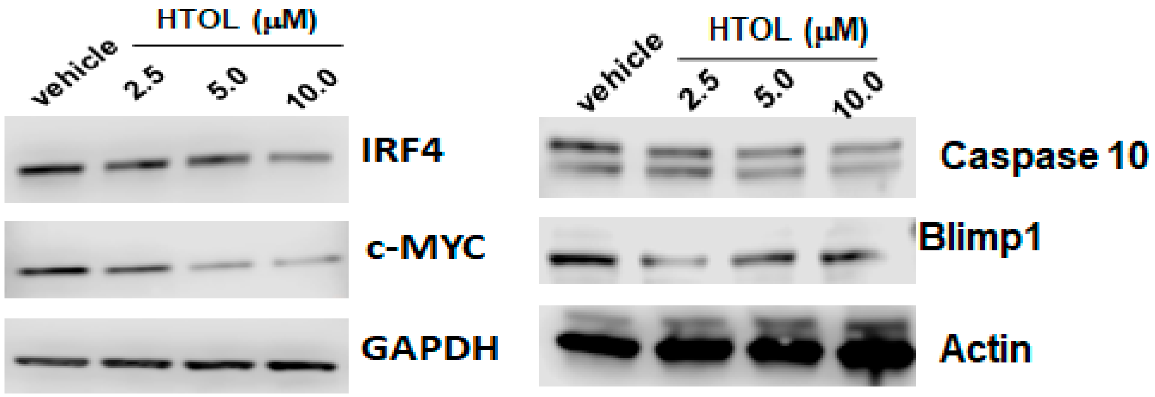

Figure S4: WB of IRF4, c-MYC, Caspase 10 and Blimp1 in NCI-H929 cells exposed for 24 h to HTOL treatment; actin or GAPDH were used as loading controls.
